# Screen time reduction and Surya Namaskars—a comprehensive intervention for nonspecific back pain in adolescents: a study protocol

**DOI:** 10.3389/fped.2025.1582984

**Published:** 2025-08-12

**Authors:** Gauri A. Oka, Ashish S. Ranade, Mayur K. Shinde, Prasad D. Pore

**Affiliations:** ^1^ Central Research and Publication Unit, Bharati Vidyapeeth Deemed University Medical College, Pune, India; ^2^ Blooming Buds Centre for Pediatric Orthopaedics, Deenanath Mangeshkar Hospital and Research Center, Pune, Maharashtra, India; ^3^ Department of Community Medicine, Bharati Vidyapeeth Deemed University Medical College, Pune, India

**Keywords:** back pain, adolescents, surya namaskars, screen time, schools

## Abstract

**Background:**

Studies have shown that excessive screen time exposure and a lack of physical exercise are associated with nonspecific back pain in adolescents. Reduced screen time exposure and Surya Namaskars (Sun Salutations) have physical and psychological benefits. We aim to test a novel comprehensive school-based intervention module—Screen time reduction and Surya Namaskars (SanSKAR) for reducing the proportion of school-going adolescents reporting nonspecific back pain. The word “sanskar” originates in Sanskrit and roughly means good values or actions.

**Methods:**

The study will be conducted in one of the six geographic divisions of a state in western India. 540 adolescent students from randomly selected urban and rural schools across five districts of this division will be included after informed assent and parental consent. Students’ heights, weights, school bag weights, and the presence and characteristics of back pain will be recorded with an on-site clinical examination by a pediatric orthopedic surgeon to rule out specific causes. Those with nonspecific back pain will perform SanSKAR: at least 12 Surya Namaskars daily at least five days a week for 16 weeks with screen time exposure of not more than 60 min per day. Post-intervention outcomes (proportion of students with back pain and its severity) will be measured at 8 weeks and 16 weeks.

**Discussion:**

This study will help test a novel multipronged school-based intervention for back pain in adolescents. The results could inform practice changes for nonspecific back pain in school-going adolescents.

**Clinical Trial Registration:**

identifier (CTRI/2024/07/070522).

## Introduction

Back pain poses an important public health challenge in today's world, not only in adults but also in children, leading to loss of academic hours, as well as personal impacts. The prevalence of back pain in children is found to be variable, with a lifetime prevalence ranging from 5% to as high as 75% (1, 2). Childhood back pain is believed to be more prevalent than previously thought, and the burden approaches that found in adults (2). Back pain in childhood predisposes the individual to the chronicity of back pain in adult life, too (3–5). Based on published literature and our own previous work in this field, back pain in the school-going adolescent age group is found to be associated with physical factors such as back injury (6), mode of transport to school, lack of engagement in sports (7), and the type and weight of the school bag, and method of carrying the school bag. Several other factors have been found to be associated with back pain, such as age, sex and BMI of the adolescent, type of school furniture and its ergonomics. Psychosocial factors such as emotional, conduct, hyperactivity, and peer problems have been found to be associated with back pain, as has the presence of a family member complaining of back pain (3, 4, 7). Screen time exposure (mobile phones, television, and laptops) is another significant contributory factor (8, 9). In India, the entire focus of all the published studies so far, except one (7), has been on the “weight of the school bag” (10–19), while others have considered postural changes associated with school bag weight (15, 20, 21). Also, in India, almost all the work in this area has been done in urban schools. There is only one study from Bhubaneshwar (22) which has included rural school-going children.

**Figure 1:**
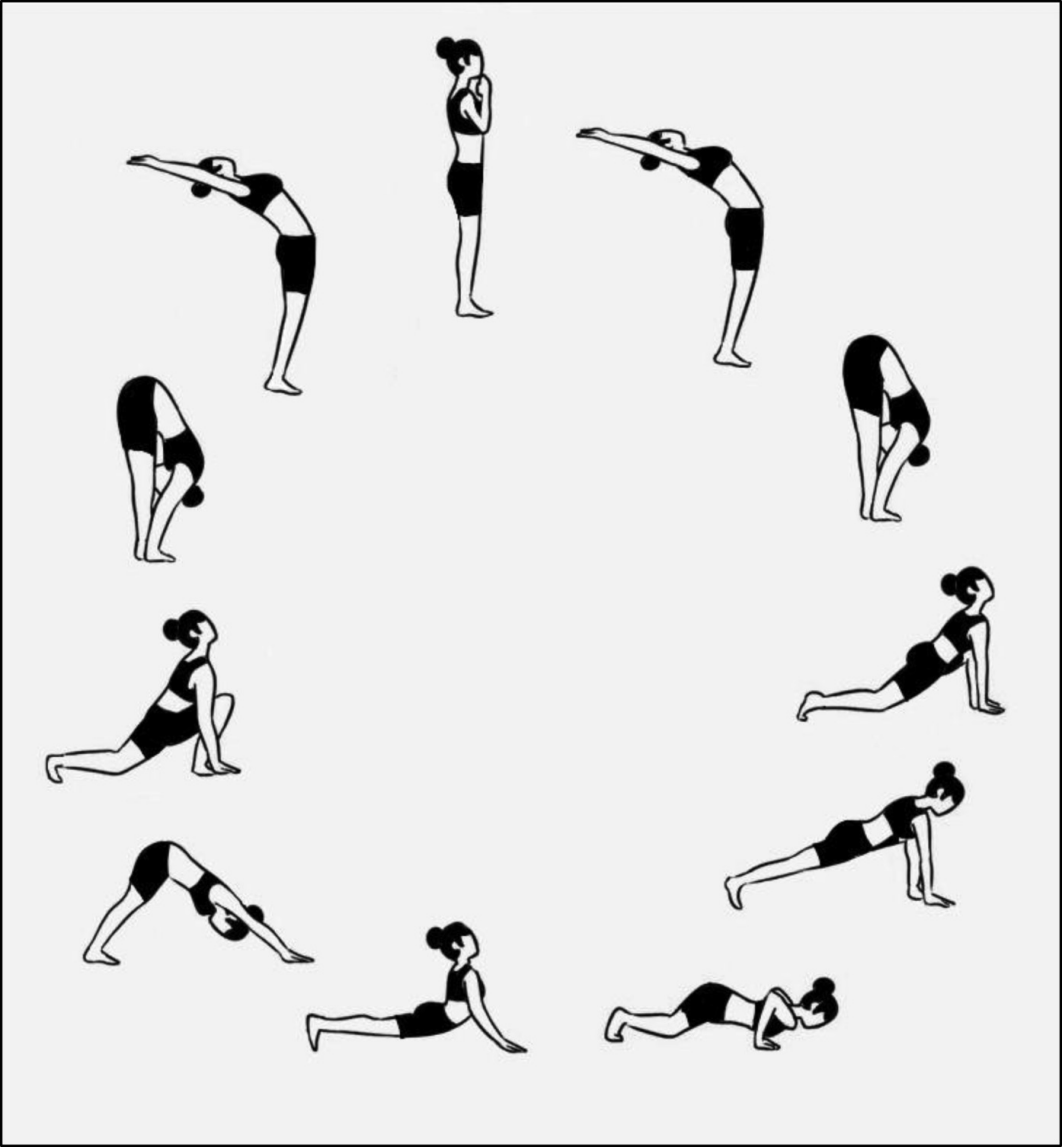
The twelve Surya Namaskar poses.

Engel's biopsychosocial model projects back pain as a complex interplay of various social, psychological, and biological factors (23, 24). The Ministry of Health and Family Welfare, Government of India, has issued operational guidelines regarding the school health program under Ayushman Bharat. They recommend “the promotion of Yoga and meditation” by incorporating the same in middle and high school curricula with a focus on “special attention to the physical, psychosocial and mental aspects” of child development (25). However, as published in a review in 2023, numerous gaps in the implementation of the holistic Ayushman Bharat school health guidelines have been discussed (26). Surya Namaskars (SN) (Figure 1) are a series of successively and continuously performed sets of 12 postures or asanas (also described as Sun Salutations) and are a component of Hatha Yoga.

SNs have been proven to enhance cardiorespiratory fitness levels, “promote weight management” (27), and be a better alternative to aerobic exercise when compared with endurance training on a stationary bicycle (28). The effect of SNs on the involved muscles using electromyography (29) has shown that SNs improve the mobility of almost all the joints of the body, with “high-to-moderate activation” of all the major truncal and lower limb muscles, concluding that SNs can be safely prescribed for not only the management but also the prevention of mechanical back pain. SNs have been shown to result in psychological benefits such as reduction in stress, anxiety, and depression, as well as improvements in emotional intelligence and quality of life in adults as well as children and adolescents (30–37).

Interventions to reduce screen time in adolescents have involved parental participation through interactive group sessions, informative flyers, posters, reminders, and maintenance of log books (38, 39). Since back pain in children is a complex conglomeration of various physical and psychosocial factors, an ideal intervention would need to be tailored to address both.

There is sufficient evidence in the currently available scientific literature to hypothesize that a structured intervention tailor-made for implementation in the school environment that includes performing Surya Namaskars and limiting screen time exposure with parent and teacher engagement could be beneficial in reducing the prevalence and intensity of non-specific back pain in adolescents. Such an intervention needs to be feasible for execution in the school environment, requiring minimal resources, and garnering student, teacher, and parent engagement so that the shared responsibility makes it more acceptable and practical.

The objectives of this study are to test a novel comprehensive intervention module “SanSKAR” (Screen time reduction and Surya Namaskars for 16 weeks) for reducing the proportion of school-going adolescents aged 10–16 years reporting moderate-to-severe back pain as well as the overall proportion of students with back pain.

## Methods and analysis

From our previous study, the proportion of students reporting moderate-to-severe back pain as measured on a VAS scale was 71.6% (7). Assuming that the intervention will cause a 15% reduction in this proportion with a 95% confidence interval and 90% power, assuming a 20% dropout rate, the calculated sample size is 270 students. To ensure sound representativeness of the results and considering the plan to conduct various sub-group analyses, this sample size will be doubled to include 540 students.

**Figure 2:**
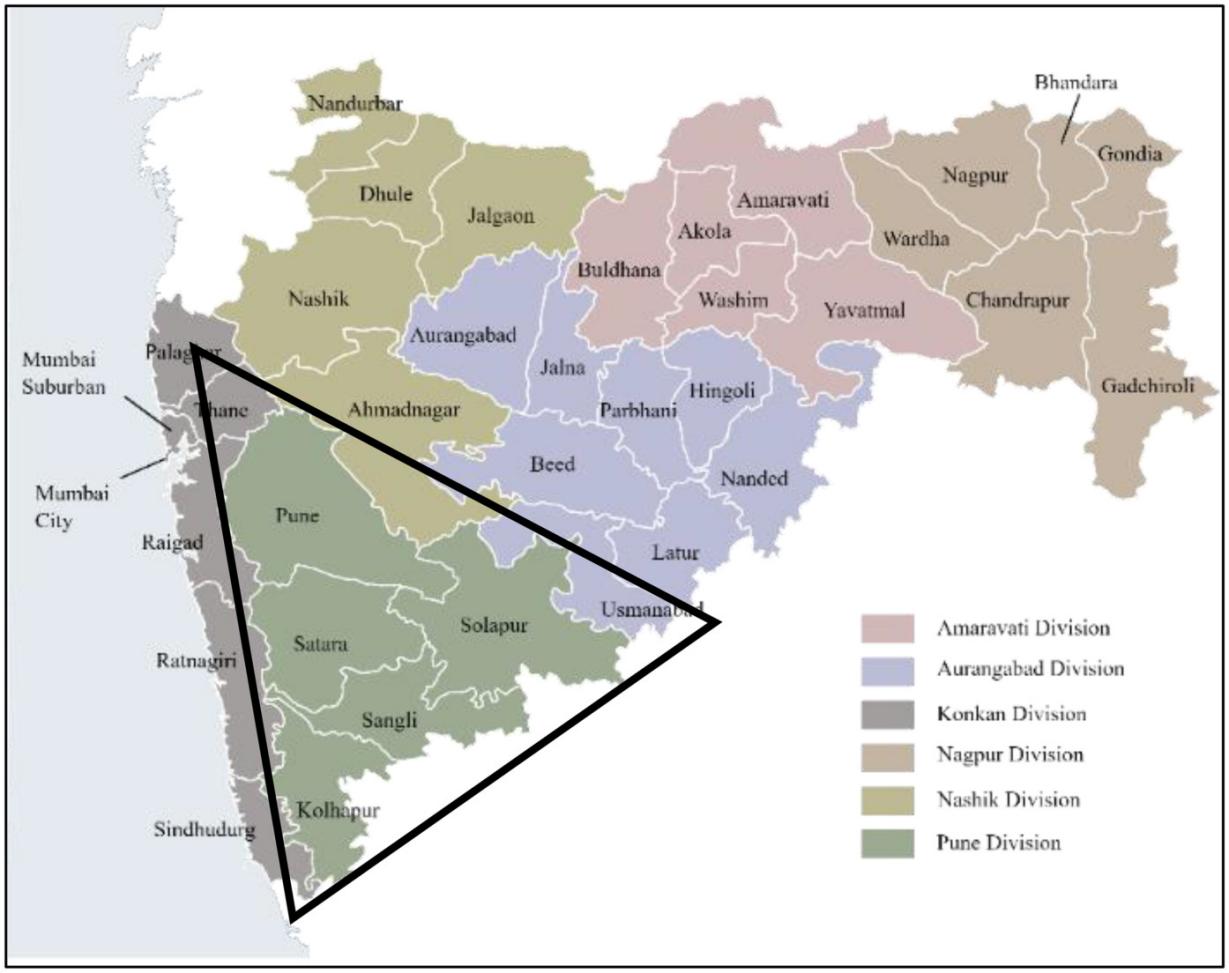
Pune division, maharashtra state (area of study marked in the black triangle).

Our sampling frame will be urban and rural schools located in each of the five districts of Pune Division, namely Pune, Satara, Sangli, Kolhapur, and Solapur (Figure 2).

Random numbers will be generated using Microsoft Excel against the lists of urban and rural schools. These numbers will be arranged in ascending order, and the first school from each pool will be approached for permission to conduct the study. If permission is refused, the next school on the list will be approached. Schools will be enrolled till the desired sample size is met. The respective school Principals will be approached for permission to conduct the study. Girls and boys studying in standards 5, 7, and 9 (who are typically between ages 10 and 16 years in Indian schools) will be enrolled as representatives of the adolescent age group if they provide written assent and parental written informed consent. Students younger than 10 years will be excluded.

This is a before-and-after study to be carried out in three phases. Phase I, where the baseline pre-intervention data will be collected; Phase II, where the intervention (SanSKAR) will be carried out on students reporting back pain; and Phase III, where the post-intervention data will be collected at the end of 8 and 16 weeks.

### Phase I

#### First visit

During the first school visit, child assent forms, parental consent forms, and printed information sheets detailing the study procedures will be distributed in the classes and sent home with the students. The project details will be discussed in detail with the respective class teachers and physical education teachers. After allowing a period of 8–10 days for responses, the date for the baseline data collection will be fixed.

#### Second visit

All the study-related procedures will be explained to the students, including height and weight measurement, school bag weight measurement, and guidelines for answering the questionnaires. A proforma will be used to document the age, sex, school grade, school bag weight, type of school bag, and students' heights and weights. A month's recall of back pain will be documented. The proforma will also include a diagram for marking the location of back pain and a visual analog scale (VAS) to note the pain severity. Details of back pain, such as frequency, duration of an episode, treatment sought, absence from school, aggravating factors, and perceived reasons, will be documented. Students will be asked to document a week's recall of the average daily time spent on mobile phones, laptops, and televisions.

Psychological data will be recorded using the self-administered Strengths and Difficulties Questionnaire (SDQ) (40) to assess prosocial behavior (strength) and hyperactivity, emotional problems, peer problems, and conduct problems (difficulties). A pediatric orthopedic surgeon will conduct an on-site clinical examination of students with back pain to identify specific causes, if any. A scoliometer examination will be performed with the student stooping forward. The scoliometer that will be used is a standard instrument developed by Dr. William Bunnell, MD (US Patent number 5181525). It is a small hand-held device used for measuirng the angle of trunk rotation (ATR). It has a notched edge and a bubble to indicate the ATR. A straight leg raise test to look for nerve root tension signs and the flexion-abduction-external rotation test to evaluate the sacroiliac joint will be done. Based on history and physical examination, imaging will be suggested if there is clinical suspicion of a specific cause of back pain. Data from Phase I will be analyzed to determine the proportion of students with back pain, its characteristics (location, intensity, frequency), and the associated physical and psychosocial factors. The SDQ scores will be computed as specified in the standard key and students will be categorized as those with low needs and those with some/high needs across the various domains.

### Phase II

#### The intervention

SanSKAR (**S**creen time reduction **an**d Surya Namas**kar**s) for students reporting back pain in Phase I.

Screen time reduction: The researcher will counsel class teachers and parents using a PowerPoint presentation and handouts detailing the intervention, including SNs and screen time reduction to not more than 60 min per day across all devices such as mobile phones, computers, laptops, tablets, and television. The counseling will include evidence-based recommendations on age-appropriate screen time exposure (41). This will be reinforced at teacher and parent engagements in weekly or fortnightly (as possible) meetings for repeated and regular advocacy and monitoring. Weekly reminders about limiting screen time exposure will be sent to the parents through the official school communication channel. The parents will be handed out a daily diary to record their child's daily screen time usage. In addition, parents will be urged to enforce a complete restriction on their child's involvement in heavy domestic or farm work (as applicable, depending upon the urban or rural setting).

#### Surya namaskars

The routine will consist of 5 min of warm-up exercises, 12 SNs with a maximum duration of 1.5 min/SN followed by *Shavasana* for a maximum of 5 min for at least 5 days a week to be continued for 16 weeks. The session is expected to last for a maximum of 30 min. Students will start the routine at least an hour after food intake, preferably in the morning. Training will be imparted to the students along with the respective class teachers and physical education instructors. Monitoring for the correctness of the technique of SNs will be continued for 5 days. Regular follow-up and monitoring of the implementation of the intervention will be done during planned school visits (minimum 8 visits and maximum 16 visits as may be practically possible) in addition to the daily monitoring by the class teachers/physical education instructors in the school.

### Phase III

Data collection will be done at the end of the 8th and 16th weeks for outcomes of interest such as the proportion of students reporting back pain, the intensity of back pain, and SDQ scores. The frequency and number of SNs performed will be noted as well as daily screen time durations. Associations if any between back pain and the physical and psychosocial factors will be determined using appropriate statistical tests. Differences in the pre- and post-intervention phases will be reported.

## Data analysis

Data will be entered in Microsoft Excel and then imported to SPSS (version 25) for Windows package (IBM SPSS Statistics for Windows, Version 25.0. Armonk, NY: IBM Corp). The associations between qualitative and quantitative variables will be determined using the chi-square test. Odds ratios will be obtained for each predictor. Multivariate regression analysis will be carried out to determine predictors of outcome variables. Pre- and post-intervention variables of interest will be analyzed by comparing their means using the paired *t*-test or comparing proportions using the McNemar test.

## Discussion

This study will help in the development of a novel multipronged school-based intervention for school-going adolescents with nonspecific back pain. All attempts will be made to refine the intervention to achieve maximum ease of administration, practicality, and acceptability to students, teachers, and parents. The experiences from this study will help inform future planning, especially from the point of view of turning this into a sustainable intervention across urban and rural schools.
